# Unveiling the Mechanisms through Which Leader Integrity Shapes Ethical Leadership Behavior: Theory of Planned Behavior Perspective

**DOI:** 10.3390/bs13110928

**Published:** 2023-11-14

**Authors:** Qaiser Mohi Ud Din, Li Zhang

**Affiliations:** Department of Business Administration, School of Management, Harbin Institute of Technology, Harbin 150001, China

**Keywords:** ethical leadership behavior, leader integrity, moral identification, self-regulation, health sector, theory of planned behavior (TPB)

## Abstract

Leadership integrity is crucial in shaping ethical leadership or promoting a rigorous adherence to moral principles and standards. This study explores the intricate relationship between leader integrity, moral identification, self-regulation, and ethical leadership behavior, providing practical insights for healthcare leaders seeking to enhance ethical practices. It addresses a critical gap in the research landscape by focusing on individual-level ethical leadership within the healthcare sector, where prior investigations have been limited. This study examines the mediating role of moral identification among leader integrity and ethical leadership behavior. We surveyed 181 health sector workers and employed SmartPLS to assess the conceptualized relationships. The analyses reveal a significant indirect influence of leader integrity on ethical leadership behavior, whereas moral identification mediates the relationship. Our findings further indicate an intriguing moderation effect of self-regulation on the relationship between moral identification and ethical leadership behavior. This divergence from previous research underscores the significance of contextual and methodological factors in studying leadership integrity and ethical behavior. Our study contributes to the literature on the relationship through planned behavior theory by demonstrating that moral identification mediates the relationship between leader integrity and ethical leadership behavior in the context of the theory of planned behavior. Our findings underscore the significance of fostering leader integrity within organizations to indirectly promote ethical leadership behaviors through moral identification. Organizations should prioritize initiatives that cultivate moral identification among their members to enhance ethical cultures.

## 1. Introduction

Leadership integrity influences ethical leadership behavior [1]. Regarding leadership, integrity is a commitment to acting morally and ethically and a consistent set of behaviors, beliefs, and values [2]. Ethical leadership behavior describes the behavior and actions of leaders that exemplify ethical beliefs, principles, and decision making [3]. Ethical leadership behavior pertains to the rigorous practice of making informed decisions and undertaking acts consistent with established moral principles and standards [4]. The study of leadership integrity and its influence on ethical leadership behavior holds significant research value, as it enhances our understanding of the significance of ethical leadership behavior within organizational contexts and its effects on diverse organizational outcomes [5].

The existing studies on leaders’ integrity and ethical leadership behavior have primarily concentrated on business organizations and political scenarios. In contrast, a limited amount of studies are available in the health sector [5]. Leader integrity and ethical leadership behavior have a significant utility where the primary function is providing ethical services and developing relative integrity for employees and the organization. The health sector faces significant challenges in ensuring integrity and ethical behavior, particularly for leaders who must make difficult organizational decisions [6]. This study also holds significance in the healthcare sector due to its potential to enhance patient care quality. Ethical leadership fosters a culture of integrity and accountability among healthcare professionals, which can lead to improved patient outcomes and safety [7]. Understanding these dynamics is critical for healthcare organizations striving to provide the highest standard of care [8]. Ethical leadership behavior requires maintaining a healthy work environment and fostering employee well-being [9].

Several scholarly studies have studied the impact of a leader’s integrity on one’s ability to lead in line with ethical principles [10,11]. A strong relationship exists between a leader’s integrity and their manifestation of ethical leadership behavior. Mayer et al. [12] conducted a study in 2009 using social cognitive theory while assessing a leader’s integrity and ethical leadership. According to numerous research investigating this relationship, moral identification bridges leaders’ integrity and ethical leadership behavior [13,14]. Research by Hsieh and Wang [15] shows that moral identification is the mediator in determining the relationship between leaders’ tendency to demonstrate ethical leadership behavior and their level of integrity. Moral identification is recognizing a person as someone who self-identifies and demands that knowledge claims about people be modest [16]. Several studies have revealed that self-regulation is vital in enhancing the connection between moral identification and ethical leadership behavior [17,18,19]. Self-regulation is the deliberate and conscious practice of controlling and modifying one’s thoughts, emotions, and behaviors, typically employed to modify reactions and behaviors [20]. Self-regulation is a moderator, suggesting that individuals with a strong moral identification are more inclined to display ethical leadership behavior.

Despite the significant progress and increasing interest in the integrity of leaders and their ethical behavior, it is evident that these ideas remain insufficiently developed and require additional investigation [1]. Furthermore, this idea has been extensively investigated within the framework of social cognitive theory [21]. Moreover, it has been examined within the setting of team dynamics, while limited study has been conducted in the domain of the health sector [5,22,23,24].

The theoretical framework used for this research is the theory of planned behavior (TPB), a psychological theory created by Ajzen [25] to forecast and modify human behavior. The TPB is a psychological framework that predicts and influences human behavior by examining three key factors: behavioral, normative, and control beliefs [26]. Using the TPB allows companies and leaders to obtain significant insights into how to foster and advance ethical leadership behaviors. Further, this research will study and investigate the relationship between individual behavior and ethical leadership behavior.

This research aims to study the correlations among leader integrity, moral identification, self-regulation, and ethical leadership behavior. The findings of this study have practical implications for leaders in the health sector who are interested in improving ethical practices within their specific environments. This research provides valuable support to ethical leadership conduct within the health sector. There has been a lack of research on ethical leadership behavior at the individual level. Unlike previous research, this study specifically focuses on the individual level. This approach is essential for multiple reasons: Firstly, by prioritizing the micro-level perspective, we can better comprehend the specific causes and dynamics that impact an individual’s behavior and decision making. Secondly, it allows us to uncover nuances and variations that might be obscured when examining data at higher levels of analysis.

Moreover, the individual-level perspective holds significant relevance in our specific context related to the health sector. This study design not only enhances the current knowledge but also provides practical insights for practitioners and policymakers aiming to tackle individual-level difficulties. Conducting research at the individual level is crucial because conflicts at the individual level might affect leadership behavior differently than conflicts at the team level. This study could provide valuable insights into the influence of organizational structures and cultural norms on the dynamics of relationships. Additionally, exploring these differences may help identify best practices for fostering effective relationships in different contexts. This study will contribute to the TPB by examining the influence of leaders’ integrity and moral identification on the development of ethical leadership behavior.

The rest of this study is divided into sections. Section 2 includes a review of the literature and the development of a theory and hypothesis. In Section 3, this article discusses the research methodology. This article describes the process and results of analyzing data in Section 4. The discussion and results of our study are given in Section 5 of this article. Section 6 discusses the practical implications of this research. Discussion of future research and this study’s limitations is covered in Section 7 of this article

## 2. Theory and Hypothesis Development

### 2.1. Theory of Planned Behavior

The theory of planned behavior (TPB) provides a framework for understanding the dynamics between leader integrity, moral identification, self-regulation, and ethical leadership behavior [26]. According to the TPB, people’s intentions and behaviors are influenced by attitudes, subjective norms, and perceived control over their behavior [27].

Within our research model, leader integrity is the independent variable that influences individuals’ moral identification, which serves as the mediator. Leader integrity reflects leaders’ ethical behavior and beliefs [23], whereas moral identification indicates an individual’s alignment with the moral standards shared by the leader or organization [28]. This alignment is shaped, in part, by the perceived social pressure and expectations regarding moral values, as represented by the “Subjective Norms” component of the theory of planned behavior (TPB) [28]. The theory of planned behavior suggests that subjective norms are crucial in shaping an individual’s moral identification [29]. These subjective norms are influenced by social influence, cultural norms, and personal beliefs, all of which contribute to forming an individual’s moral compass. Additionally, research [30] has shown that when leaders demonstrate high levels of integrity it positively impacts the moral identification of their followers, leading to increased ethical behavior within the organization.

Furthermore, self-regulation serves as a crucial moderator in this framework. It demonstrates an individual’s ability to regulate actions, explicitly upholding moral beliefs [20]. The concept of control, referred to as perceived behavioral control in the theory of planned behavior (TPB), influences the connection between moral identification and ethical leadership conduct, our dependent variable [31]. Individuals who perceive themselves as having control over their ethical behaviors are more inclined to exhibit ethical leadership behavior [32]. In contrast, a lack of perceived control may have a negative impact on ethical leadership behaviors. Behavioral intentions, part of the theory of planned behavior (TPB), are intimately associated with ethical leadership behavior [31]. They reflect individuals’ goals to demonstrate ethical leadership activities. Strong behavioral intentions indicate a higher probability of engaging in ethical leadership conduct, as intentions substantially impact actions. However, it is essential to note that behavioral intentions alone may not always translate into actual ethical leadership behavior. Various situational factors and external pressures can impact an individual’s ability to act in accordance with their intentions. Therefore, fostering a supportive organizational culture and providing resources for ethical decision making can further enhance the likelihood of ethical leadership behavior.

Using the TPB framework to understand the factors influencing ethical leadership behavior, our research investigates the complex connection between leader integrity, moral identity, self-regulation, and behavioral intentions. The impact of a leader’s integrity on an individual’s moral identification is influenced by social norms and is moderated by self-regulation. This ultimately affects the individual’s intention and actual display of ethical leadership behavior, providing a comprehensive understanding of the factors involved in ethical leadership behavior.

### 2.2. Ethical Leadership Behavior and Leader’s Integrity

Leadership integrity is a crucial component of leadership that has not been widely explored with voice. This refers to its influence on leadership behavior and how that impact can be seen [1]. Moorman et al. [33] argue against the concept that leader integrity and ethical leadership behavior must be considered the same. Leader integrity describes the personal beliefs and integrity of a leader. In contrast, ethical leadership behavior refers to a leader’s acts and behaviors that align with ethical principles and values [34]. Leader integrity is a fundamental characteristic of ethical leader behavior. It refers to consistently aligning actions, values, methods, measures, principles, expectations, and outcomes [1]. This entails a strong dedication to doing what is morally right, regardless of the situation. Leader integrity is crucial for establishing trust and credibility among followers [2]. It sets the foundation for ethical behavior and is a role model for others to emulate. When leaders consistently demonstrate integrity, it creates a positive organizational culture that promotes ethical decision making and fosters a sense of fairness and justice [5]. The measures and definitions of leader integrity have experienced changes over time. However, similar to ethical leadership research, the research of leader integrity has faced challenges due to a lack of clarity in its theoretical foundation [21]. The study of leader integrity within the health sector holds significance due to its role in building trust, facilitating ethical decision making, cultivating a strong organizational culture, and strengthening the reputation and credibility of health organizations [6,35,36].

Martin and Colleagues [37] explain ethical leadership conduct as reflecting moral principles, values, and integrity in making decisions and engaging in relationships. Ethical leaders aim to establish an organizational climate characterized by trust, respect, and ethical accountability, thereby fostering ethical behavior among employees. The organizational culture involves long-term values and beliefs, whereas the organizational climate pertains to the current environment and the leader’s behavior and perceptions [38]. Both concepts are crucial in establishing the work environment and impacting a leader’s behavior and well-being. According to Zappla and Toscano [39] ethical leadership behavior can be a dynamic development in which leaders adeptly integrate ethical beliefs, virtues, and principles into their behaviors and decision-making procedures. This involves exemplifying ethical behavior and motivating others to keep to ethical principles. The organization seeks to establish a setting that emphasizes transparency, accountability, and integrity, thereby contributing to the organization’s ethical culture as a whole.

Multiple research studies consistently show that the existence of leader integrity has a positive impact on ethical leadership [1,5,40,41]. This is mainly attributed to leaders who exhibit integrity, serve as role models for their followers, and encourage them to follow ethical standards and be involved. Nevertheless, the specific process through which a leader’s integrity contributes to developing ethical leadership behavior remains unclear. One potential mediator that might clarify this relationship is moral identification, which refers to how individuals define themselves in connection to their moral beliefs and ideals. Still, it is essential to note that there may be dissimilarities in how different persons respond to the effect of leader integrity and moral identification on ethical leadership.

Thus, it is hypothesized that the direct effect of leader integrity on ethical leadership will be more substantial for individuals with higher levels of consistency. Individuals with more consistency may be more adept at internalizing and translating integrity into ethical leadership behavior. In contrast, individuals with less consistency in behavior in their leadership practices may have a weaker indirect effect.

**Hypothesis** **1.** *Leader integrity has a significant direct impact on ethical leadership.*

### 2.3. Moral Identification

Moral identification can be described as the extent to which an individual’s sense of oneself is influenced by the importance of being moral [42]. But, if individuals view moral qualities such as honesty, compassion, fairness, and generosity as crucial in shaping their identification, they possess a robust moral identification. According to theory, people with strong moral identities act more morally [42,43,44]. While leader integrity refers to a leader’s constant commitment to a moral or ethical code, moral identity relates to how people view and assess themselves and others based on ethical beliefs [45]. Both moral identities and leader integrity play crucial roles in shaping individuals’ behaviors and decision-making processes [46]. While moral identification focuses on personal values and beliefs, leader integrity emphasizes the importance of leaders serving as ethical role models for their followers [47]. By embodying and upholding moral principles, leaders with high integrity can inspire trust, promote ethical behavior, and foster a positive organizational culture [48]. Investigating moral identification within the health sector contributes to healthcare workers’ ability to adhere to ethical principles. This study contributes to avoiding moral distress, cultivating trust, and promoting the well-being of leaders and healthcare professionals [49].

The moral identification of individuals within an organization is positively influenced by the level of integrity shown by a leader [50]. Moral identification describes a person’s engagement with a particular set of moral principles and ideals that direct their ethical behavior [44,51]. The main focus lies in examining the significance of leaders and their integrity in potentially shaping the moral identification of their subordinates or followers. The proposition posits that leaders who constantly exhibit elevated levels of integrity, marked by ethical conduct, honesty, and transparent decision making, are more inclined to cultivate a heightened sense of moral affiliation among their subordinates. As a result, these employees are likely to make moral decisions aligned with the organization’s ethical standards, exhibit ethical behavior, and feel a greater sense of organizational commitment. Thus, the following is hypothesized:

**Hypothesis** **2.** *Leader integrity positively influences moral identification.*

Moral identification refers to an individual’s alignment with ethical values and principles, which can influence their decision making and actions within the organization [52]. In contrast, ethical leadership behavior involves actively exemplifying these values by setting a positive example, promoting fairness, and holding others accountable for their behavior [53]. By combining moral identification and ethical leadership behavior, leaders can create a culture that encourages ethical conduct at all levels of the organization [54].

Additionally, the key focus of this research is to examine the potential mediating effect of moral identification on the correlation between leader integrity and ethical leadership. This finding implies that the extent to which followers identify morally can affect how leader integrity is perceived with ethical leadership. The correlation between leader integrity and ethical leadership is expected to be influenced by the moral identification of followers [55]. When individuals possess a greater degree of moral identification, the effect of leadership behavior linked to integrity on their perception of ethical leadership becomes significantly greater [56].

**Hypothesis** **2a.** *Moral identification mediates the relationship between leader integrity and ethical leadership.*

### 2.4. Self-Regulation

According to the social cognitive perspective, self-regulation is an ongoing relationship between three key factors: personal, environmental processes, and behavioral [57]. Furthermore, self-regulation, which refers to an individual’s capability to control and regulate their thoughts, emotions, and actions, has been recognized as a fundamental characteristic of ethical leadership behavior [20,58]. Ethical leadership behavior pertains to the actions and behaviors exhibited by leaders to foster ethical behavior within their teams or organizations [59]. Self-regulation affects an individual’s behavior, while ethical leadership affects the overall ethical climate and the behavior of others inside the organization [60]. Self-regulation and ethical leadership conduct are crucial for cultivating an ethical culture [61]. Self-regulation emphasizes individual accountability, while ethical leadership behavior highlights the role of leaders in establishing an ethical environment [62]. According to Salaxiddinovna et al. [19], individuals with higher self-regulation levels demonstrate enhanced abilities to withstand temptation, make ethical decisions, and consistently exhibit ethical behaviors over an extended period.

Achieving organizational success and facilitating behavioral change holds significant importance across diverse contexts. Examining self-regulation within the health sector is essential in facilitating staff well-being, mitigating turnover rates, and improving patient care. The implementation of specific strategies aids in the establishment of a work environment that promotes well-being and enhances productivity, resulting in favorable outcomes for both individuals and businesses [63]. Therefore, it is hypothesized that the positive correlation between moral identification and ethical leadership will be more prominent among individuals with high self-regulation abilities. Conversely, individuals with lower levels of self-regulation may exhibit a weaker or non-significant association between moral identification and ethical leadership as they may struggle with consistently translating their moral values into leadership actions.

**Hypothesis** **3.** *Self-regulation moderates the relationship between moral identification and ethical leadership, such that the positive relationship is stronger for individuals with higher levels of self-regulation.*

**Figure 1:**
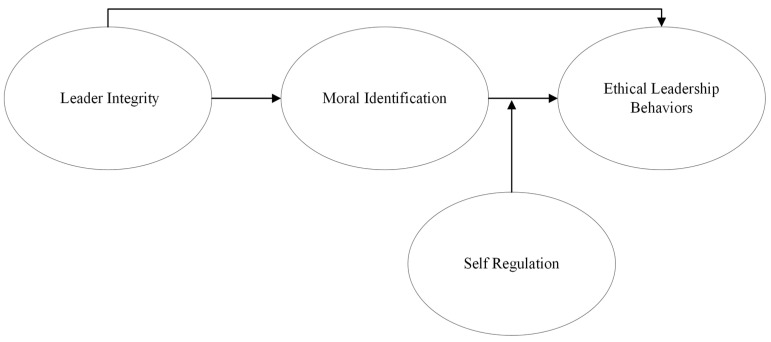
Conceptual framework of this study.

The conceptual framework for this article is presented below see Figure 1.

## 3. Research Methodology

The sampling frame for our study was defined to encompass the healthcare sector in Harbin, Heilongjiang Province, China. We selected Harbin due to its significance as a central regional healthcare hub, representing a diverse range of hospitals and healthcare facilities. We chose Harbin as our research location because it provided an easily accessible setting for our study. Harbin City was explicitly selected for multiple reasons. Firstly, it is one of the most influential cities in Heilongjiang Province with an established healthcare infrastructure. Secondly, the city boasts a range of private healthcare institutions, which offers us a wealth of data to analyze. Lastly, Harbin’s healthcare sector reflects the trends and challenges within the healthcare system, making it an ideal location for our research. Our study focused on healthcare professionals working in hospitals in Harbin; this included doctors, nurses, administrative staff, and other healthcare team members. By concentrating on this group of professionals, we aimed to gain insights into their perspectives and experiences within the healthcare sector, which are crucial aspects for understanding ethical leadership and integrity dynamics within this context.

We utilized data from an unknown population in our research due to the absence of precise population size data for the healthcare sector in Harbin, Heilongjiang Province. This approach aligns with established survey research principles, as Groves et al. [64] acknowledged, emphasizing the importance of rigorous sampling and statistical analysis when dealing with unavailable population information. This research collected data from the healthcare sector in Harbin, Heilongjiang Province, which includes multiple hospitals. A comprehensive survey was conducted, wherein a sample of 11 distinct hospitals in Harbin were contacted. A total of 350 questionnaires were distributed, of which 223 were returned, resulting in a response rate of 57.71%. Among the returned questionnaires, 180 were deemed useable. The data were gathered over the period spanning from May 2023 to August 2023.

The gender distribution revealed that 73.3% of the participants were female and 26.7% were male. Regarding age, the participants predominantly fell within the 25–40 category. Regarding education, 26.1% of participants held an MBBS Degree, while 31.1% had completed nursing education, 25% had attained a health-related degree, and 17% specified another educational qualification. Lastly, participants’ work experience was categorized into different categories, with the highest percentage falling within the 6–10 years range. This brief presentation offers a snapshot of the demographic composition of this study’s participants within the research methodology section.

A rating system comprising anchors ranging from “strongly agree” to “strongly disagree” has been adopted. The leader integrity scale comprised a total of five components. We adapted the leader integrity scale devised by Moorman et al. [33] to assess leader integrity. The items are adapted from two dimensions of the leader integrity scale, behavioral integrity and moral behavior, with 14 items outlined by Moorman and colleagues [33]. The scale includes items that assess various aspects of leader integrity, such as honesty, ethical behavior, and consistency between words and actions. By adapting this scale, we aimed to capture the multidimensional nature of leader integrity and ensure its validity in our specific research context. The moral identification scale’s development was derived from the seminal research conducted by Reed and Aquino [65], which has a 13-item scale to measure moral identification. This scale was designed to effectively measure an individual’s level of moral identification, providing valuable insights into their ethical beliefs and behaviors. The moral identification scale comprised a total of six items. The measurement of self-regulation was performed utilizing a scale containing 5 items, which was adapted from the work of Neal and Carey [66], which has 63 items. We only adapted five items from Brown et al.’s self-regulation questionnaire [67], which are more closely related to self-regulation behavior.

The selected items were chosen based on their relevance to the specific context of our study and their demonstrated validity and reliability in previous research. The Ethical Leadership Behavior Scale was developed based on the research conducted by Kalshoven et al. [68]. The Ethical Leadership Behavior Scale comprised a total of four items. To measure ethical leadership behavior, we used the Ethical Leadership behavior questionnaire developed by Kalshoven and colleagues [68], which contains 46 items from 7 sections. The questionnaire comprises the sections listed by Kalshoven et al. [68]: fairness, ethical guidance, role clarification, and people orientations. Each section consists of items that assess specific aspects of ethical leadership behavior. The questionnaire has been widely used in previous research to evaluate the ethical leadership practices of individuals in various organizational settings. The order of the items in the questionnaire was randomized to avoid bias in further factor analyses.

By reducing time and facilitating cross-validation of the results, a validated questionnaire can improve the survey’s validity and reliability. The decision to selectively incorporate a limited number of items from the original questionnaire for each construct was made to maintain our survey’s conciseness, specificity, and applicability to our study objectives. Given the limitations of respondent time and the potential for survey fatigue, a shorter questionnaire is more likely to yield higher response rates and more thoughtful responses [69]. These items were selected based on their relevance to our research focus and objectives. The initial version of the questionnaire was created in English and subsequently translated into Chinese, the official national language, to facilitate its administration to local participants.

Consequently, the questionnaire was translated according to the established translation criteria [70]. A language communication specialist translated it into Chinese. This procedure aimed to identify and align the translated items with the original English version of the questionnaire to provide an accurate Chinese translation [71].

Our research used the Smart-PLS 4 software to apply the structural equation modeling (SEM) technique. We extensively validated these tools to ensure their reliability. We also conducted SEM investigations to explore and evaluate the proposed hypothesis. Path modeling, an employed technique in management research, helps predict cause–effect relationship models [72]. According to Leguina [73], Partial Least Squares Structural Equation Modeling (PLS-SEM) has proven effective in generating solutions for models that involve constructs, indicators, and structural links. Hair et al. [74] suggest that PLS-SEM is suitable for theory development and evaluation. This methodology enables the examination of constructions and exploration of connections within models of interest in the relationship between ethical leadership behavior and leader integrity. Partial Least Squares Structural Equation Modeling (PLS-SEM) is a statistical analysis method that provides benefits. It excels at handling sample sizes and effectively managing models and does not rely on strict assumptions about data distribution. According to Hair and colleagues [75], PLS-SEM is particularly effective in accommodating models without needing a sample size or making assumptions about data distribution. According to Leguina [73], in the context of Partial Least Squares Structural Equation Modeling (PLS-SEM), it is recommended that the sample size be at least ten times higher than the number of arrows directed toward a construct. PLS-SEM is deemed highly appropriate for the current study.

## 4. Data Analysis and Results

### 4.1. Reliability and Validity Analysis

**Figure 2:**
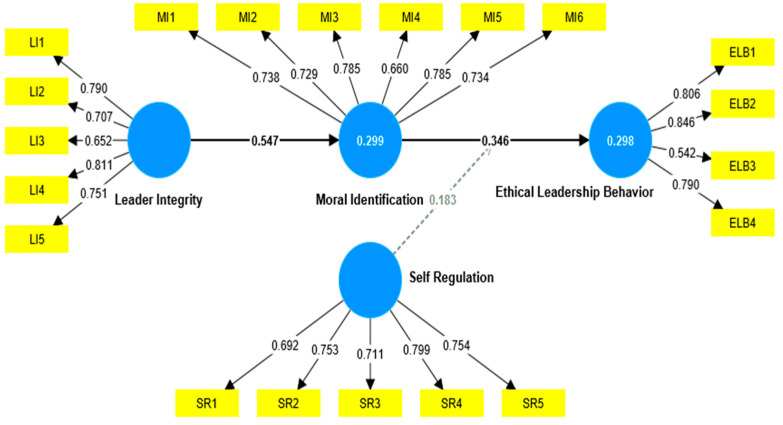
Graphical output PLS-SEM.

The measurement model was assessed to determine the reliability and validity of the constructs, as shown in Table 1 and graphical Figure 2. According to Hair et al. [76], all the model items exhibit loadings higher than the least acceptable threshold of 0.50.

While it is generally preferred to have factor loadings over 0.7 [77], it is common for researchers in the field of social science to observe lower outer loadings (<0.70) in their investigations, removing indicators on composite reliability, content validity, and convergent validity instead of employing an automatic elimination approach. Following the guidelines recommended by Hair et al. [74], it is generally recommended to investigate removing items with outer loadings ranging from 0.40 to 0.70. However, this removal should only be pursued if eliminating such items improves the AVE or composite reliability outside the suggested threshold. In the present investigation, excluding the item (ELB3, loading = 0.542) would not have substantially improved the AVE and composite reliability measures, as the construct’s values already exceeded the required level. Moreover, the assessment of the confidence interval for the loadings in the current study indicates that no loadings in the items include a value of zero. Consequently, no items were excluded from further analysis in the study.

The examination of reliability was carried out using Cronbach’s alpha, rho_C, and composite reliability measures, all of which provided results exceeding the required threshold of 0.700 [78]. The obtained rho_c value fell within the range, Sarstedt et al. [79] reported for composite reliability and Cronbach’s alpha. Furthermore, the value exceeded 0.70, suggesting a high level of reliability, as indicated by Henseler et al. [80]. The AVE exceeding the threshold of 0.500 demonstrated the acceptability of convergent validity. The discriminant validity testing involved comparing correlations among the latent variables using the heterotrait–monotrait ratio of correlations Henseler and Colleagues [80], with values below the conservative threshold of 0.85. Therefore, discriminant validity may be established. (See Table 2).

Variance inflation factors (VIFs) are a comprehensive analytic tool for assessing collinearity and offer a significantly superior alternative to examining simple correlation values [81]. The VIFs have a significant advantage in that they indicate which coefficients are affected by collinearity, as Michael and colleagues showed [82]. Typically, when the variance inflation factor (VIF) for a variable (βi) is less than 10, it suggests that there is no multicollinearity present [83]. Nevertheless, Diamantopoulos and Siguaw [84] introduced a stricter criterion stating that the variance inflation factor (VIF) for βi should be less than 3.3. Table 3 illustrates that all the values shown are below the established threshold of 3.3. Common method variance (CMV) poses a significant risk to survey-based studies, mainly when relying on a single respondent [85]. To assess CMV, several studies in the field of strategic management recommend Harman’s Single-Factor test [86,87,88]. With this method, we entered all the items of the variables into a single-factor test in SPSS. We found that the first factor only explained 37% of the variation, which is less than the cutoff ratio of 50%. Hence, we confirmed that there is no problem with CMV in the dataset. In addition, we performed the complete collinearity variance inflation factors (VIFs) test, as suggested by Kock [89], to detect the presence of CMV. The author recommends using a VIF threshold of 3.3 in CMV testing when employing factor-based PLS-SEM algorithms. Table 3 displays the whole VIF value for each construct. It is clear that these values are below 3.3, indicating that the proposed research model is free of CMV [90].

### 4.2. Structural Model

**Figure 3:**
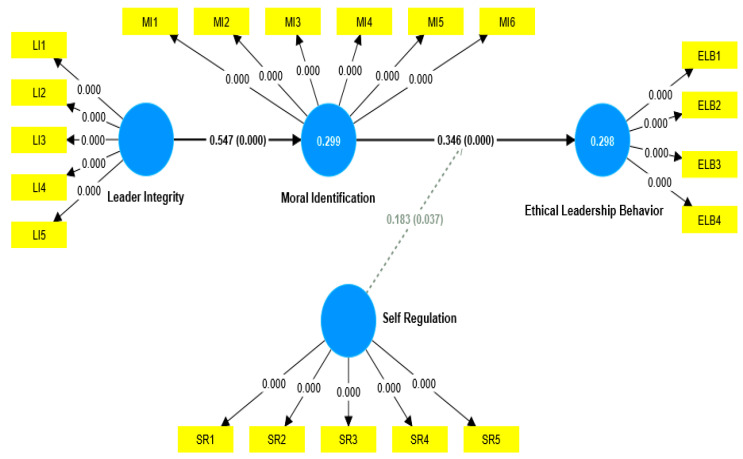
Graphical output structural model.

The hypotheses proposed in the study are reflected in the structural model. Following bootstrapping in structural analysis, Figure 3 displays the graphical representation of path coefficients and *p*-values.

A structural model is evaluated by considering many metrics, including the Q^2^, R^2^, and significance of paths. These paths encompass specific direct and indirect effects. The model’s predictive power is assessed based on the significance of each structural path, as indicated by the R^2^ value for the dependent variable [91]. According to Falk and Miller [92], the R^2^ value should ideally equal or exceed 0.1. The findings presented in Table 3 indicate that the R^2^ values exceed 0.1. Therefore, the ability to make predictions has been established.

Moreover, Q^2^ serves to establish the predictive significance of the constructs. A positive value for Q^2^ indicates that the model possesses predictive validity. The results suggest a statistically significant relationship in the prediction of the constructs, as shown in Table 4.

To assess the significance of the relationship in the framework, hypotheses were examined as part of a subsequent analysis to evaluate the validity of the model’s fit. This research aims to investigate the possible direct impact of leaders’ integrity on ethical leadership behavior. This study’s results suggest that leader integrity has a notable direct impact on ethical leadership behavior (β = 0.189, t = 3.580, *p* < 0.001). Consequently, the hypothesis H1 was supported. This finding supports the idea that leaders with high levels of integrity are likelier to engage in ethical leadership behaviors. This result aligns with previous research [2] that has emphasized the importance of leader integrity in promoting ethical conduct within organizations.

According to this study, a leader’s integrity significantly affects moral identification (β = 0.547, t = 0.069, *p* < 0.001). The hypothesis H2 is supported. This result is consistent with the previous study by Ete, Z et al. [93], which found a positive relationship between a leader’s integrity and followers’ moral identity. These findings suggest that leaders with high levels of integrity are likelier to foster a strong sense of moral identity among their followers.

According to this study’s findings, moral identity was a significant mediator as this was this study’s primary objective (β = 0.189, t = 3.580, *p* < 0.001). The study results indicate a correlation between moral identification and a leader’s ethical behavior. This discovery supports hypothesis H2a, which is inconsistent with the previous study by Gerpott and his colleagues [94], which found no significant relationship between moral identity and ethical behavior. These findings suggest that moral identity plays a crucial role in shaping a leader’s ethical conduct, contradicting previous research.

Additionally, the research findings suggest that self-regulation significantly moderates the relationship between moral identification and ethical leadership behavior (β = 0.183, t = 2.081, *p* = 0.019). Despite the *p*-value for the effect approaching 0.50, it still provides evidence for the influence of self-regulation in this context. The outcomes of hypothesis testing can be seen in Table 4. The significant beta coefficient (β = 0.183) and t-value (t = 2.081) indicate a meaningful association between these variables, reinforcing the notion that self-regulation is a crucial factor in shaping ethical leadership behavior.

## 5. Discussion

The findings of our analysis align with the theoretical frameworks proposed in prior research, which identified a favorable correlation between leader integrity and ethical leadership conduct [2]. Furthermore, they confirm the importance of leadership integrity in promoting ethical behavior within organizations. This research also adds to the existing literature by highlighting the role of leadership integrity in fostering a positive ethical climate and influencing employee behavior. Additionally, it emphasizes the need for organizations to prioritize leader integrity as a critical factor in promoting ethical conduct throughout the workplace. Furthermore, our analysis indicates that leader integrity significantly impacts moral identification, which aligns with the findings of a prior study conducted by Ete, Z et al. [93]. This study demonstrated a positive correlation between a leader’s integrity and the moral identity of their followers. These results imply that followers of leaders with high integrity are more likely to develop a strong sense of moral identity. This strong sense of moral identity can lead to increased ethical behavior and decision making within the organization. Furthermore, this study also highlights the importance of leaders serving as role models for their followers, as their integrity can significantly impact their subordinates’ moral values and behaviors.

Previous research has shown that moral identification mediates between leader integrity and ethical leadership, aligning with investigations [20]. The main goal of this study was to analyze how leader integrity indirectly affects ethical leadership behavior via moral identification. The study results indicate a correlation between moral identification and a leader’s ethical behavior. This result is inconsistent with the previous study by Gerpott et al. [94], which found no significant relationship between moral identity and ethical behavior. However, it is essential to note that Gerpott and his colleagues [94] focused on a different aspect of ethical behavior, an adherence to rules and regulations.

In contrast, the current study specifically examined moral identification. Therefore, these contrasting findings suggest that various factors may influence ethical behavior and it cannot be solely attributed to moral identity alone. Further research is needed to explore the complex relationship between leader integrity, moral identification, and ethical leadership behavior.

Our study reveals a finding regarding how self-regulation affects the link between moral identification and ethical leadership conduct. The significant *p*-value of 0.019 supports this conclusion, contrasting with studies that reported a more pronounced moderating effect [19,20]. This finding suggests that self-regulation plays a smaller role in moderating the relationship between moral identification and ethical leadership behavior than previous studies. It is vital for future research to explore further the factors that may influence this relationship and examine other potential moderators that could have a more substantial impact. It is crucial to consider factors contributing to this difference, such as unique organizational or cultural aspects, within our study’s context [95].

Furthermore, it is worth noting that variations in sample characteristics, measuring instruments, or statistical techniques could potentially account for the observed difference [96]. We acknowledge the importance of exploring factors contributing to this difference, including unique organizational and cultural aspects within our study’s context and potential variations in sample characteristics, measuring instruments, or statistical techniques. Additionally, considering the possible influence of external factors, such as socioeconomic status or geographical location, could provide further insights into the observed differences.

These results are in accordance with the individual-level framework known as the theory of planned behavior [25,97]. According to the TPB, perceptions of behavioral control, subjective norms, and attitudes affect intents and behaviors. Within our research, leader integrity is vital in influencing attitudes and subjective norms connected with ethical leadership. Moral identification, functioning as a mediator, includes the attitudes and norms that subsequently impact ethical leadership behavior. The moderating function of self-regulation aligns with the concept of perceived behavioral control in the TPB. This study suggests that persons with higher levels of self-regulation are more capable of effectively translating their moral identification into actions correlated with ethical leadership. Furthermore, individuals with strong self-regulation skills are more likely to resist external pressures and adhere to their ethical principles, thus reinforcing their ethical leadership behavior. Additionally, research has shown that leaders who exhibit ethical behavior gain the trust and respect of their followers and create a positive organizational culture that promotes ethical decision making at all levels.

Understanding how leader integrity affects a person’s moral identification might help to explain how this aligns with the TPB [65,98]. The moral identification of individuals is firmly rooted in their self-concept, significantly affecting their intentions and behavior [65,99]. The relationship strengthens through self-regulation, improving individuals’ capacity to align their actions with their moral identification and reinforcing their perceived behavioral control [98,100]. This alignment between leader integrity and moral identification can also increase followers’ trust and loyalty. Furthermore, research has shown that individuals strongly identifying with their moral values are more likely to engage in ethical decision making and exhibit prosocial behavior.

In conclusion, our study shows the complex relationships among leader integrity, moral identification, self-regulation, and ethical leadership behavior. Although our findings deviate from previous studies regarding the moderation effect of self-regulation, they emphasize the significance of considering contextual and methodological aspects. The relationship between our results and the TPB enhances our comprehension of the fundamental processes that link leader integrity and ethical leadership behavior. These findings suggest that leader integrity is crucial in shaping ethical leadership behavior. Moral identification and self-regulation may not be this relationship’s mediators or moderators. Understanding these findings’ contextual and methodological aspects can help organizations develop effective strategies for promoting ethical leadership within their ranks.

## 6. Implications for Practice

This research highlights the importance of sustaining leader integrity throughout organizations. The demonstration of integrity by leaders can indirectly promote ethical leadership behaviors among their subordinates through moral identification. Organizations must focus on measures that foster moral identification among their personnel, including facilitating a robust ethical culture and values. The moderating influence of self-regulation on the correlation between moral identification and ethical leadership shows the significance of individual diversity. Organizations must recognize the inherent variability in individuals’ self-regulatory capacities and customize their leadership development initiatives to accommodate these differences.

Organizations should emphasize developing ethical decision-making abilities, promoting the capacity to assess complex scenarios and make ethically appropriate decisions. Organizations may promote ethical behavior and improve the capabilities of their employees by providing them with the necessary information and skills for ethical leadership. This will result in a more ethical, resilient, and efficient organization. Organizations exhibit their dedication to ethical values and guarantee that their workers have the necessary tools and knowledge to lead with integrity by proactively investing in ethical leadership training and development. Organizations must prioritize accountability and openness in their pursuit of ethical leadership. This involves creating transparent processes for reporting and resolving ethical issues or violations. Ensuring that all individuals within the organization understand the consequences of engaging in unethical conduct and have secure channels to report such behavior is essential. Organizations should proactively communicate their priority for ethical leadership behavior and the penalties for misbehavior. This method serves as a preventive measure against unethical behavior and a reinforcement of the significance of ethical behavior inside the organization. In addition, providing clear and open communication about ethical issues and ensuring that they are addressed effectively helps build confidence and trust among employees and stakeholders, strengthening the organization’s reputation for ethical leadership.

The alignment of these findings with the TPB provides a theoretical framework for understanding and predicting ethical leadership behavior. Organizations can use the TPB to inform their leadership training programs and interventions, focusing on subjective norms, attitudes, and perceived behavioral control. Organizations can shape employees’ attitudes by cultivating a culture that admires ethical behavior and developing the notion that ethical leadership is admirable and highly regarded. Leadership role modeling and communication efforts can shape subjective norms by highlighting the organization’s prevailing ethical leadership behavior. Ultimately, perceived behavioral control can be increased by offering individuals the necessary tools, resources, and assistance to engage in ethical decision making effectively.

The divergence in the moderation effect from prior research underscores the need to consider contextual and methodological factors in organizational studies. Researchers and practitioners should be cautious about generalizing findings across different contexts and populations. This study highlights the intricate interplay of variables in ethical leadership, emphasizing that it is not a one-size-fits-all concept. When developing ethical leadership initiatives and policies, organizations should heed this complexity, considering the nuanced interplay of variables within different demographic and professional roles. This understanding is crucial, as it allows organizations to tailor their ethical leadership initiatives to meet the unique needs and challenges of diverse individuals and groups. By recognizing the intricate nature of ethical leadership and considering the specific dynamics within different demographic and professional roles, organizations can foster a culture of inclusivity and fairness that promotes ethical behavior across all levels. Organizations should encourage continuous learning and adaptation in leadership and ethics. This includes updating the latest research findings and adapting leadership practices accordingly. These implications provide actionable insights for organizations seeking to promote ethical leadership and enhance the impact of leader integrity within their ranks. Organizations foster a culture of continuous innovation by remaining aware of the most recent research insights and adapting leadership approaches accordingly. This proactive strategy guarantees that ethical leadership efforts stay pertinent and efficient in tackling developing ethical concerns across various demographic and professional positions.

## 7. Limitations and Future Research Direction

One limitation of our study is that it relied on cross-sectional data. Future research could benefit from longitudinal designs to establish causal relationships and track changes in ethical leadership behavior over time. This study used a specific sample from the health sector of China, and its generalizability to different industries, cultures, or organizational contexts may be limited. Future research should aim to replicate these findings across different settings to improve external validity. Since all variables in this study were self-reported, common method bias may be a concern. Employing objective measures or alternative data sources can help mitigate this limitation. The moderation effect of self-regulation was unexpectedly different from prior research. Investigating the factors contributing to this variability in moderation effects, such as individual differences or contextual factors, should be a focus of future research.

Future research should conduct longitudinal studies to explore how leader integrity, moral identification, self-regulation, and ethical leadership behavior evolve. This study can provide insights into the dynamics of ethical leadership development. Future work can also examine how these relationships differ across various organizations and cultures. Cultural differences may influence the strength and nature of these associations. Future research based on experimental designs should establish causality and test interventions to enhance leader integrity and moral identification to promote ethical leadership. Objective measures, such as behavioral observations or peer evaluations, should be incorporated to complement self-reported data and reduce common method bias.

Further, other potential moderators of the relationship between moral identification and ethical leadership should be explored to better understand the conditions under which this relationship is strengthened or weakened. Future researchers can develop and test interventions to enhance self-regulation skills in leaders to promote ethical leadership behaviors effectively. Future research could attempt to use social cognitive theory and the theory of planned behavior (TPB) to improve our comprehension of the psychological foundations of ethical leadership behavior. This comparative investigation will provide insight into the complex mechanisms that impact ethical leadership behavior.

Moreover, we intend to investigate the influence of the ethical climate inside an organization on the growth of leader integrity, moral identification, and ethical leadership. This research will provide valuable insights into the broader organizational context, clarifying the complex dynamics. These future research directions can help expand our knowledge of ethical leadership, its antecedents, and its outcomes, contributing to more effective leadership practices and ethical organizational cultures.

Researchers incorporating qualitative methodologies can also better understand individual behavior and ethical leadership. This mixed-methods approach will allow researchers to offer more nuanced insights and strengthen the practical implications of our findings. By integrating qualitative methodologies, we aim to capture individuals’ subjective experiences and perspectives, providing a richer context for our quantitative data with different demographics. This holistic approach will enable us to explore the interplay between individual behavior, ethical leadership, and the broader organizational dynamics that influence them.

## Figures and Tables

**Table 1:** Validity and reliability analysis.

Constructs	Item	Loadings	α	Rho_C	AVE
Leader integrity	LI1	0.790	0.800	0.861	0.554
	LI2	0.707			
	LI3	0.652			
	LI4	0.811			
	LI5	0.751			
Moral identification	MI1	0.738	0.835	0.879	0.547
	MI2	0.729			
	MI3	0.785			
	MI4	0.660			
	MI5	0.785			
	MI6	0.734			
Self-regulations	SR1	0.692	0.797	0.860	0.551
	SR2	0.753			
	SR3	0.711			
	SR4	0.799			
	SR5	0.754			
Ethical leadership behavior	ELB1	0.806	0.750	0.838	0.571
	ELB2	0.846			
	ELB3	0.542			
	ELB4	0.790			

**Note:** Rho_C, composite reliability; AVE, average variance extracted; α, Cronbach’s Alpha.

**Table 2:** Heterotrait—monotrait ratio discriminant validity.

	ELB	LI	MI	SR
Ethical leadership behavior				
Leader integrity	0.308			
Moral identification	0.514	0.639		
Self-regulations	0.542	0.482	0.548	
Self-regulations × moral identification	0.056	0.220	0.318	0.359

**Table 3:** Collinearity statistics (VIF).

LI- > MI	1.000
MI- > ELB	1.311
SR- > ELB	1.333
SRx MI- > ELB	1.148

Note: LI, leader integrity; ELB, ethical leadership behavior; MI, moral identification; SR, self-regulation; VIF, variance inflation factor.

**Table 4:** Hypotheses testing.

Hypotheses	β	SD	t	P	Q^2^	R^2^	Results
LI- > ELB	0.189	0.053	3.580	0.000	0.201	0.286	Supported
LI- > MI	0.547	0.069	7.963	0.000	0.269	0.295	Supported
LI- > MI- > ELB	0.189	0.053	3.580	0.000			Supported
SR × MI- > ELB	0.183	0.088	2.081	0.019			Supported

Note: β, Beta coefficient; SD, standard deviation; t, t-statistics; P, *p*-value; Q^2^, Q-square value, R^2^, R-square value; LI, leader integrity; ELB, ethical leadership behavior; MI, moral identification; SR, self-regulation.

## Data Availability

Data sharing is not applicable to this article. The dataset associated with this research is not publicly available due to the privacy and confidentiality commitments made to the study participants. Ensuring the protection of respondent privacy was of utmost importance in this research, and as such, the raw data cannot be made openly accessible.
